# Supplementary material for: Encoding of visual stimuli and behavioral goals in distinct anatomical areas of monkey ventrolateral prefrontal cortex
Source: PLoS Biol. 2025 Aug 7;23(8):e3003041. doi: 10.1371/journal.pbio.3003041 (PMC12342307; doi:10.1371/journal.pbio.3003041)
Supplement: S1 Table — (PDF) [file pbio.3003041.s001.pdf]

Table 1

Cases	Species	Hemisphere	Area	Injection number	Tracer	Amount
23	Fascicularis	Left	Middle 46v (Skeletomor network)[a]	9	FB 3%	2 × 0.2 µl
26	Nemestrina	Left	45B (Oculomotor network)[a]	38	DY 2%	1 × 0.2 µl
			Caudal 12r (Oculomotor network)[a]	29	FB 3%	1 × 0.2 µl
30	Nemestrina	Right	45B (Oculomotor network)[a]	35	FB 3%	1 × 0.2 µl
36	Fascicularis	Left	45B (Oculomotor network)[a]	39	FB 3%	1 × 0.2 µl
			45A (Oculomotor network)[a]	34	CTBg 1%	1 × 1 µl
		Right	8FEF (Oculomotor network)[a]	16	FR 10%	1 × 1 µl
			45B (Oculomotor network)[a]	37	BDA 10%	1 × 2 µl
37	Rhesus	Left	45A (Oculomotor network)[a]	31	FB 3%	2 × 0.2 µl
			8r (Oculomotor network)[a]	15	CTBr 1%	1 × 1 µl
			8FEF (Oculomotor network)[a]	17	CTBg 1%	1 × 1 µl
		Right	45A (Oculomotor network)[a]	33	BDA 10%	2 × 1 µl
			45B (Oculomotor network)[a]	36	FR 10%	1 × 1 µl
39	Fascicularis	Left	45A (Oculomotor network)[a]	32	FR 10%	2 × 1 µl
43	Rhesus	Left	Rostral 46v (Intraprefrontal)[b]	1	CTBr 1%	1 × 1 µl
			Middle12r (Skeletomor network)[b]	25	FB 3%	1 × 0.2 µl
			Middle 12r (Skeletomor network)[b]	24	DY 2%	1 × 0.2 µl
		Right	Caudal 46v (Oculomotor network)[b]	14	FR 10%	1 × 1 µl
44	Rhesus	Left	Caudal 46v (Oculomotor network)[b]	13	DY 2%	1 × 0.2 µl
			Middle 46v (Skeletomor network)[b]	6	FB 3%	1 × 0.2 µl
		Right	Middle 12r (Skeletomor network)[c]	26	LYD 10%	1 × 1.3 µl
			Middle 12r (Skeletomor network)[c]	27	FR 10%	1 × 1 µl
47	Rhesus	Left	Caudal 12r/Middle12r (Oculomotor/Skeletomor network)[c]	28	LYD 10%	1 × 1.3 µl
			Rostral 12r (Intraprefrontal)[c]	21	BDA 10%	1 × 2 µl
			Rostral 12r (Intraprefrontal)[c]	18	FR 10%	1 × 1 µl
		Right	Rostral 12r (Intraprefrontal)[c]	19	FB 3%	1 × 0.2 µl
			Middle 12r/Rostral 12r (Skeletomor network/ Intraprefrontal)[c]	23	DY 2%	1 × 0.2 µl
48	Rhesus	Left	Caudal 12r (Oculomotor network)[c]	30	LYD 10%	1 × 1.3 µl
		Right	Middle 12r/Rostral 12r (Skeletomor network/ Intraprefrontal)[c]	22	DY 2%	1 × 0.2 µl
			Rostral 12r (Intraprefrontal)[c]	20	FB 3%	1 × 0.2 µl
51	Rhesus	Left	Middle 46v (Skeletomor network)[b]	11	DY 2%	1 × 0.2 µl
			Middle 46v (Skeletomor network)[b]	10	FB 3%	1 × 0.2 µl
			Rostral 46v (Intraprefrontal)[b]	2	BDA 10%	1 × 2 µl
		Right	Rostral 46v (Intraprefrontal)[b]	3	CTBg 1%	1 × 1 µl
52	Rhesus	Left	Middle 46v (Skeletomor network)[b]	7	FB 3%	1 × 0.2 µl
			Middle 46v (Skeletomor network)[b]	5	DY 2%	1 × 0.2 µl
		Right	Middle 46v (Skeletomor network)[b]	8	LYD 10%	1 × 1.3 µl
			Caudal 46v/Middle 46v (Oculomotor/Skeletomor network)[b]	12	FR 10%	1 × 1 µl
			Middle 46v (Skeletomor network)[b]	4	BDA 10%	1 × 2 µl

Cases from [a] Gerbella et al. (2010); [b] Gerbella et al. (2013); [c] Borra et al. (2011).
