# Supplementary material for: Encoding of visual stimuli and behavioral goals in distinct anatomical areas of monkey ventrolateral prefrontal cortex
Source: PLoS Biol. 2025 Aug 7;23(8):e3003041. doi: 10.1371/journal.pbio.3003041 (PMC12342307; doi:10.1371/journal.pbio.3003041)
Supplement: Attachment — Submitted filename: response to reviewers.docx [file pbio.3003041.s011.docx]

**Responses to Reviewers.**

**Reviewer #1**

*The manuscript is built on top of the lab's previous expertise regarding the anatomical characterization of the frontal cortex and the paper's topic. The analyses are sound, combining classical and modern approaches at single cell and population levels, which collectively support the conclusions drawn by the authors. The statistical significances appropriately take into account the effect of multiple comparisons over time when that occurs (decoding analysis). The manuscript is well-written and clear, and the results are original and well-contextualized in the previous literature. The study limitations are clearly stated and transparently addressed. Finally, the involvement and specialization of prefrontal cortex components during the goal-directed behavior conveyed by the active task is of important interest. Therefore, for these reasons, I recommend the publication of the manuscript.I do not have any major comments. But only some minor comments (listed in order of presentation in the manuscript).*

We thank the reviewer for her\his positive comments. We addressed all the minor points below.

*-Line 178-180 (Figure legend 2): It is not clear to me what "above" and "below" mean. Shouldn't they refer to the bar graphs shown and thus be "left" and "right"?*

Figure and caption have been modified.

*-Line 182 (Figure legend 2): Red squares.*

Figure and caption have been modified.

*-Line 232: Typo, remove "for".*

Done.

*-Line 265-266: Typo, instead of "Figure 2K," it should be Figure 4K.*

Done

*-Line 419-421: If I have not misunderstood, the use of "specific activity pattern" might be confusing for the reader, since (as also stated immediately after) cross-temporal decoding in S11B and S12B shows stability between epochs of presentation, decision, and behavioral response, suggesting a shared activity pattern between epochs.*

The text has been corrected accordingly.

*-Line 757: It is written "video," but it should be "image".*

Done.

*-Line 835-842: Although this information may have been reported in previous publications using this dataset, I suggest including in this manuscript the number of electrodes used (or an average estimate) to obtain the neuronal signal in the recorded sessions. In order for the reader to have a general understanding of how many neurons could be recorded simultaneously.*

We added some details in the Methods section. In particular, in the new version of the manuscript it is now clarified that we recorded from a maximum of 8 electrodes per session and that for each electrode we recorded the activity from 3 depths.

*-Line 1042: Although it is stated that decoding was implemented as described in previous publications using this dataset, I would also include some key details in this manuscript for clarity. Specifically, it would be helpful to specify the type of classifier used, the method for cross validating the data and the percentage/number of test trials used, any data normalization procedures applied, and whether pseudo-population techniques were employed to account for non-simultaneously recorded neuronal populations.*

We added further details in the Methods section, better explaining how the decoding analysis was performed.

*-Line 1045-1047: This definition may lead to confusion and potentially conflict with the classification of static time points derived from the analysis of the cross-temporal decoding matrices. As described in the Methods section, an off-diagonal time point is classified as static if the difference in accuracy from the on-diagonal time points used for training and testing is not statistically significant. For this assessment, it is only considered that the accuracy of the off-diagonal time point is significantly greater than chance without considering its absolute magnitude value.*

We better defined static pattern in the Methods and Results sections.

*Line 1061-1065: Part of the method described here involves the first steps of the statistical calculation performed by the cluster-based permutation test. I would move this description a little later when the statistical test is explained in detail.*

Done

*-Fig 4c: In this figure, unlike the others, the number of neurons is reported instead of percentages. Unless there is a specific reason, I would also use percentages here as well to maintain consistency across the figures.*

*-Fig 4n: There is a typo on the y-axis labels.*

*-Figure legend 4: A description of Figure 4C is missing.*

The figure has been modified according to the reviewer’s suggestions. Now histograms are no longer displayed, since in some cases the number of neurons of a single monkey (e.g. selective responses to videos) was too small and did not reach the sampling criteria for a solid statistical comparison among tasks and properties (see also response to reviewer 2).

*-Figure S10-11: Considering that the variable "condition" and "object" lead to two different chance levels in the decoding analysis (50% and 33% accuracy, respectively), I would suggest adding a horizontal line in the plots regarding "Significant on-diagonal time bins" to make explicit for the reader the expected chance level.*

Figures have been removed, but the significant bins are now directly outlined on figures 9 and S7.

**Reviewer #2:**

*The strength of the study comes from their ability to identify distinct anatomical areas across the two monkeys based on a previous anatomical parcellation, their ability to nicely condense a rich amount of results and that they were able to have the two monkeys preform three different tasks. However, it should be noted that the diversity of the tasks could have been further exploited by not choosing two passive visual tasks but instead an auditory task for example as the similarity of the two passive visual tasks limits the conclusions that can be drawn from the study. Also, the sample size is quite low (n=2 monkeys) and the number of trials for each task not that high (sometimes per stimulus just around 10 trials were performed).*

The reviewer perfectly centered the general idea of the study: mapping the largest possible number of neuronal basic properties. Indeed, we also performed an auditory task (Rozzi et al., 2011, SFN proceedings 914.10/QQ15), but, because of some technical limitations, we could not produce data solid enough to be included in the present paper. The task had the same structure of the other passive tasks, but instead of videoclips or images, 3 different sounds (pure tone, peanut cracking, monkey coo-call) were played-back with a high frequency (96kHZ) loudspeaker located behind the screen, positioned in front of the monkey. Since the auditory task was the easiest among the employed tasks, due to its short duration, we usually ran it as the last one. The temporal position of this task, added to the fact that we recorded neurons in acute, led us to record a lower number of neurons compared to those recorded in other tasks, thus resulting in a less robust sample of data for our objective of comparing the properties of the different VLPF areas.

Concerning the sample size, two monkeys are required for sake of reproducibility and usually considered acceptable, in the scientific literature, for neurophysiological studies, because ethical reasons lead to a limitation in the number of employed non-human primates. In fact, statistical tests are not performed on subjects, but on the single neuron activity recorded in the different trials. Ten trials is the number most often (almost always) used in literature.

*My main concern is that for most of the analyses it was not clear whether they have pooled data over the two monkeys. I think it is crucial to demonstrate how robust results are across the two monkeys or whether results are driven by a single monkey. Already in Fig. 2 it became clear that results are not that consistent across monkeys and I would have wished for more insights into Fig. 2C/F and 3 C/F on a single monkey level: Do both monkeys show consistency in the identified regions?*

*For many remaining figures (Fig. 5. And Fig. 6), it was not even clear whether these were results from combined monkey data or just a single monkey selected.*

We decided to remove the histograms from the figures, since they could be misleading. In fact, in some cases the number of neurons recorded in a single monkey (e.g. selective responses to videos) is too small and does not reach the sampling criteria for a solid statistical comparison of the number of neurons among tasks, properties and subjects. The differences between monkeys are evident from the maps (presented in the manuscript separately for the two monkeys) and now described in more detail in the Results and in the Discussion sections.

In addition, to better describe similarities and differences between the two monkeys, we made further analyses and modified the structure of the Results section. Specifically, we ran a clustering analysis on the firing rate of VLPF neurons. This analysis has the advantage of considering the bin-by-bin firing rate throughout the whole task, providing a more general view on neuronal activity. By applying the clustering procedure on different functional groups of neurons, we were able to show both functional similarities and differences between monkeys (see Results and response to reviewer 4, for more details). Based on these results, we validated the anatomical map on each monkey, supporting the fact that population analyses are performed on the data of the two monkeys pooled together.

We also ran population analyses separately on each monkey, finding consistency in the results (see below, as an example, the decoding analysis on the Condition factor).

Finally, in the Results section, we explicitly stated that population analyses are performed on the data combined from the two monkeys.

*Another drawback is that most of their results center around caudal and middle 12r/46v and conclusions about the functional specialization of rostral sections of VLPFC and 45A are thus very limited.*

Even though the sample of the rostral sector and area 45A was more limited with respect to the other areas due to the dimensions and shape of the recording chambers, the number of recorded neurons in these sectors was sufficient to run analyses that allowed us to differentiate them from the neighboring ones; therefore, we left their description in the Results and in the Discussion section.

My other points are:

*- In the parcellation section of the results and methods it is mentioned that areas were also identified based on how differentially connected they are. To me it was not clear how this was achieved? Also given that their parcellation is so central to their study, I feel some more details should already be provided in the Results section about that.*

We better described the parcellation method in the Methods and Results sections. In addition, we included a table in the Supplementary Materials, listing all cases used for performing this parcellation.

*- Is there a way to validate the parcellation? Given that Fig 2. And Fig. 3 already show little consistency between the monkeys, could one reason be that the anatomical parcellation actually does not translate well to a functional parcellation?*

*- I come from a human fMRI research background, where we expect large inter-subject variability in the functional topography of frontal regions and networks, even among healthy young adults. Is such variability not assumed in monkeys, particularly in frontal regions? How can we confidently conclude that the same anatomical region in different monkey's maps to the same functional region? Is inter-subject variability in functional organization not expected in monkeys?*

As mentioned above, the connectional data used for the parcellation comes from 20 hemispheres of 12 monkeys, thus they are quite robust to be generalized. In addition, in order to address this comment and a similar one by reviewer 4, we performed a cluster analysis that, as described above, allowed us to functionally validate our anatomical parcellation.

Regarding the second point raised by the reviewer, although we expect some inter-subject variability, the temporal and spatial resolution of single unit recording enabled us to be in the best conditions to find common properties across the monkeys. Following the reviewer’s comment, we now also describe the functional differences between the two monkeys, by considering the maps and the cluster analysis.

*- Why are different number of neurons recorded for each task?*

As already described above, the differences in number of neurons recorded in each task are mainly due to the recording technique employed in this work. Indeed, by recording neurons in acute during sessions with long durations, in which the monkeys performed the various tasks in a sequence, the neurons recorded by each electrode during each task could be lost in time, resulting in a different number of neurons recorded in the single sessions across tasks.

*- In the result section, it was not clear what the 2 X 12 ANOVA (Epochs, Stimuli), 3x6 ANOVA, and 9x2 ANOVA were referring to. It would help to briefly explain the different epochs defined in the main text for each task (e.g. for pictures 2 epochs: baseline and fixation and 12 different stimuli presented). In the video task description, there is also a mention of epoch 2 that the reader is not able to understand without knowing how these epochs are defined.*

We now describe, also in the Result section, the type of factors and epochs of the different ANOVAs we carried out.

*- Fig. 2 and 3: In the Figure legends, the reference to above and below does not seem correct (should be left and right I assume).*

Figures 2 and 3 and the relative captions have been now updated to respond to the first major point of the reviewer.

*- Fig. 5: To inspect the suppression better plotting a zero line would be helpful.*

Done

*- Fig. 6: Is there a way to combine some of the figures from the supplementary into this figure? I feel it would already be helpful to see above threshold clusters in Fig. 6.*

We changed the figure following the reviewer’s suggestion. Now the significant bins are outlined in red in the main figure.

*In addition to above-chance stable coding clusters, it may also be interesting to look at dynamic coding clusters?*

Cross-temporal decoding analysis on neuronal firing rate is usually employed to verify whether the same type of code is present in different time periods, and thus if the population of neurons encode different events in the same way. Thus, statistical tests currently used to validate this analysis assess significant and reliable decoding effects related to a “maintenance” of accuracy levels when training and testing on different time periods. Implicitly, this means that when no static pattern is present, the type of neural code changes. Though in principle interesting, it is very difficult to discuss a non-significant finding (dynamic pattern).

*- I feel the manuscript could benefit from a summary figure that relates findings to different anatomical regions.*

We added a final figure (fig. 10) summarizing the location of functional properties.

Discussion:

*- How does the anticipation of rewards may impact the passive visual viewing task results? Would results differ if no rewards would have been given*

In principle, part of the neural responses could be explained in terms of expectation of reward, but, in monkey studies, the reward is necessary to assure the correct performance of the behavioral paradigm. However, it is commonly accepted that using the same amount and type of reward for all stimuli and conditions, as in our tasks, does not differentially influence the observed responses.

Methods:

*- The distinction between goal-related and non-goal-related actions in videos and images was not explored. Wouldn't incorporating this dimension enhance the explanatory power of the analysis and help strengthen the conclusions of the paper?*

The issue of neuronal selectivity for specific groups of videos and the possible impact of the request to actually categorize stimuli has been the focus of a previous paper by our group, and this is now cited in the Discussion section.

*- P 31, line 757: This should be images and not videos in the sentence.*

Corrected.

*- The input to dPCA is not well described; authors referred to an existing method but the reader should to understand the data matrix that entered the dPCA.*

We introduced a new paragraph, in the Methods section, in which the data matrix used for population analysis is described in more detail.

**Reviewer #3:**

*1. My main comment is that the manuscript would benefit from a clearer articulation of its main aim and how it builds on previous publications using the same dataset. The authors state that a key goal is to assess congruence between their anatomical parcellations—based on cytoarchitecture and connectivity— and functional properties. However, this does not feel like a central focus in the current version, and the manuscript lacks a strong link between the uniqueness of the parcellation and the functional findings. The introduction should better motivate why this specific parcellation is advantageous—not just by calling it "more refined," but by explaining what new insights it enables and why it's important to categorize the electrophysiological data this way. Relatedly, the manuscript would benefit from a clear hypothesis. What does the parcellation predict about the ephys results? Much of the discussion feels post-hoc and confirmatory of previous findings; if the analysis is exploratory, that should be stated explicitly. If there are a priori hypotheses, they should be clearly introduced up front.*

We thank the reviewer for her/his constructive comments which allowed us to improve the quality of our paper.

In the present version of the manuscript, we largely rewrote the Introduction, to better explain the rationale and the hypotheses.

In the revised version, we first briefly review the functional and anatomical literature showing some type of inhomogeneity along the VLPF. Then, we frame the functional literature in the light of different models (as suggested by the reviewer in the following point). Finally, we clearly state the aim of the study, that is to “map the distribution of VLPF neuronal properties based on the hypothesis that functional specialization depends on the characterizing connections of different anatomical sectors”.

Even if concise, the descriptions of the anatomical connections provided should now clarify that there are strong functional hypotheses based on the connectional patterns. For example, we predict a stronger presence of visual responses in the ventral areas more connected with inferotemporal region, and of motor-related responses in the middle sector, strongly connected with the parietal and premotor hand regions. This should also clarify the choice to compare the data based on the anatomical parcellation.

Finally, note that in the Discussion section these hypotheses are now more clearly discussed.

*2. The manuscript is heavily framed around a rostro-caudal organizational gradient of the LPFC. While this framework typically spans the full extent of the LPFC—from premotor areas to the frontal pole—the current results seem to reveal a much narrower gradient within the mid-VLPFC. Perhaps the authors may wish to consider alternative organizational schemes that might better account for their findings. For example, the multiple-demand framework (Crittenden & Duncan, 2012, Cerebral Cortex; Kadohisa et al, 2023 Neuron; Assem et al 2020 Cerebral Cortex) offers a contrasting view, challenging strict hierarchical models.*

We thank the reviewer because this criticism allowed us to deepen the theoretical discussion of our data. We have now reduced the emphasis on the rostro-caudal gradient in both the Introduction and Discussion sections and have incorporated considerations on the multiple-demand framework and adaptive coding models. These models indeed better account for our finding, allowing us to highlight in the Introduction and to thoroughly discuss in the Discussion the evidence that some functional features are “broadly distributed”, while others are “more localized”. This point is now addressed in detail, also with specific reference to the results of the analysis conducted in response to the second major comment raised by Reviewer 4.

*3. Correct me if I'm misunderstanding the task, but in the visuo-motor condition, it seems like the monkey can already decide whether to act or not during the cue period (based on the red or green cue). If that's the case, could this early decision-making explain the "static coding" observed across areas—like what's shown in the condition decodings of Figure 6? I'm just trying to reconcile this with prior work showing more dynamic coding in similar regions (e.g., Stokes et al., 2013, Neuron). Some discussion of how this task structure might influence representational dynamics would be helpful.*

In the Discussion section we now address how the structure of our task could influence the static pattern observed. Note that the result is not the same in all areas, suggesting that the effect is more due to the functional properties of the specific area than to the type of task. There are differences and similarities between Stoke’s work (now appropriately cited) and the present one, related to both the regions and the phases of the task in which a static pattern is observed. In particular, our cues allow to accurately predict the general goal (action/inaction) that the monkey will have to achieve in the subsequent phases of the task. Especially, in the Inaction condition, the cue allows the monkey to “fully” program the behavior to perform (not acting). The “predictive” nature of cue information in our task structure could be one of the reasons possibly leading to the generalization found with the decoding analysis between initial and final phases, for example in areas such as middle 12r (as also discussed in Rozzi et al., 2023).

In Stoke’s work, Go and NoGo information is not conveyed by Cue appearance, but is defined by the subsequent stimulus (Target) which has to be correctly classified based on the a-priori rule as “Go” or “NoGo”. Since in this latter work the saccade direction is already predictable, based on the location of the presented cue, we hypothesize that a stable code representing “saccade direction” information would be found between the Cue/Target and the period in which the saccades are actually executed (not considered in the task). Note, finally, that the areas deeply involved in predictive coding in our study are only marginally explored in their study.

*4. The decoding analysis looks solid overall, and the steps are well explained—but I'm a bit concerned about the statistically significant small off-diagonal clusters that show up during baseline in several areas (supp figures). Some of these are about the same size as the clusters the authors base key interpretations on. It's not clear why these baseline clusters survive if the analysis is properly controlled. Maybe it's worth double-checking the stats—do you need a stricter correction? Possibly more permutations or a higher cluster threshold to rule out spurious effects?*

We agree with the reviewer that the mentioned effect can appear to be “puzzling”. However, the statistical requirements we adopted are already extremely strict (the most conservative we found in literature) and we are concerned that further increasing them will hide solid and interesting results. We believe, however, that there is a simple explanation for these small clusters of significant bins observed in the baseline period. Our baselines are taken from the intertrial, and trials are presented in a randomized order. Thus, the monkeys cannot, in principle, predict whether the next trial will belong to one condition or the other. However, we observed that, in some sessions, the monkeys adopted a behavioral strategy that could possibly allow them to predict the Condition of the next trial, during the baseline period. We ran a behavioral analysis, considering all sessions in which task-related neurons were recorded (n=174). We found that in 6% of the sessions (n=11) the monkeys performed more than 2/3 of the Inaction trials in the first AND more than 2/3 of the Action trial in the second part of the session, suggesting that they executed the task as if it were in blocks: in the initial part of the session they correctly performed most of the trials belonging to the Inaction condition (which are easier), while generally neglecting Action trials. This strategy led them to perform many Action trials consecutively towards the end of the sessions (in fact the incorrect trials were recovered at the end to obtain the required number of correct trials for each condition). In this situation, it is thus possible that, at some point of the session, the monkeys already expected/knew what the next trial was going to be.

We believe that, though very sporadic, this strategy could account for the effect observed by the reviewer.

Note, finally, that the observed “baseline” effect is actually related to very small clusters of consecutive bins, and in the revised version of the manuscript we decided to focus only on larger clusters.

*5. Minor Corrections and Clarifications:*

** Figure 1 caption: correct "cito-architecture" to "cytoarchitecture."*

Done.

** Improve figure 2 caption*

** Figure 4 caption: currently missing explanation for panel C.*

The figures 2 and 4 have been changed to respond to the comments of other reviewers. Thus, the caption of these figures has been now updated also to reflect this suggestion.

** Figure 5, Middle 12r plot during the object presentation phase has a mistake (avg line is in a different location than the error shades).*

Corrected

**Reviewer #4:**

*Major points:*

*- The current analyses could benefit from additional approaches that capture neuronal activity more comprehensively. Specifically, a geometrical analysis could help disentangle task-related variables in a more intuitive and interpretable manner (e.g. see approaches to separate sensory from choice/action information by decoding axes as in Mante et al, Nature 2013). The reliance on ANOVA alone is insufficient, as its hand-selected factors do not adequately account for the high dimensionality of the neuronal representations that are typical of the PFC.*

We thank the reviewer for the suggestion. In the revised version we used an approach similar to that employed by Mante et al., running a demixed Principal Component Analysis to provide a more comprehensive description of the neural dynamics associated to each task variable. Figures 5, 6 and 7 depict the neural dynamics emerging when projecting the neural activity onto the subspace defined jointly by components related to the considered factors and by components capturing factor-independent variability. In the new version, we emphasized these results and thoroughly discussed them.

*- An unsupervised clustering analysis of neuronal response profiles should be performed to assess whether functional clusters emerge that align with known anatomical parcellation and connectivity. The current analysis groups neurons into pre-defined anatomical regions. I would like to know whether grouping by function produces similar results. Functional boundaries need not necessarily respect anatomical boundaries.*

We thank the reviewer for this suggestion that allowed us to increase the solidity of our data and to enrich our discussion. We performed a functional parcellation to validate the anatomical maps based on connectional data. This analysis, now presented in the main manuscript and in the Suppl Mat, reveals that specific properties are clustered in regions defined by connectional parcellation, thus indicating the presence of a match between functional and anatomical borders.

*- The visuo-motor task results are described in qualitative terms without numerical quantifications. In general, no statistical tests are provided to assess whether neuronal responses are unevenly distributed across recording sites. If the authors claim a spatial organization of function within the VLPFC, they should include a test for non-uniform spatial distribution to support this conclusion. Listing (small) differences in percentages is not sufficient.*

In the new version of the manuscript, we removed the quantitative description of the distribution of functional properties, since numbers could mislead the reader and it is not possible to use robust statistics since the sample is, in most cases, not large enough. For example, the number of selective responses to videos is too small and does not reach the sampling criteria for a solid statistical comparison of the number of neurons among areas.

However, we maintained the qualitative description of the functional maps in order to allow the reader to make her/his own evaluation of the homogeneity or inhomogeneity of the distribution. Furthermore, we added the above described unsupervised analysis to support the presence of a differential spatial distribution of some functional properties.

*- The descriptions of experimental tasks in the main text are too rudimentary, and merely referring to the Methods section is insufficient. A brief but comprehensive summary should be provided in the main text to ensure readers can follow the rationale and design without constantly needing to refer back to the Methods.*

The behavioral paradigms are now described in more detail in the initial part of the Results section.

*- Likewise, several methodological aspects are not properly explained. Some examples are*

*"mean-net activity" (l. 303) appears to involve only baseline subtraction but does not specify whether additional normalization steps (e.g., division by variance) were applied. Clarification is needed.*

In this analysis, we only performed baseline subtraction without further normalization. This is now clarified in the Results and Methods section.

*- "injection" is ambiguous (e.g. l. 126) - does it refer to current injection for electrolytic lesions or something else?*

The term the reviewer is referring to indicates the injection of neural tracers. This has been clarified in the description of how the parcellation has been achieved, which is now reported in more detail in the Results and Methods section.

- *the type of decoding analysis (e.g., LDA, SVM etc) is not described in the Methods section*

The methods section now includes further details on how the decoding analysis was performed, including the type of classifier (Poisson-Naïve Bayes).

*Minor points:*

*- Were the data sets newly acquired for this study or re-used from previous work? If existing data were analyzed, the motivation statement in the last paragraph of the Introduction should be adjusted accordingly to avoid overstating the novelty of the study.*

The dataset analyzed in this work was already partially employed in previous works, not aimed to define the areal distribution of properties. We now changed part of the Introduction section, including the aim of the study, to address both this point and the reviewer’s 2 comments.

*- There is a sudden shift in style starting on p. 16 (decoding results), where findings are discussed by anatomical area rather than by task or analysis, as was done in the previous sections. The authors should maintain a consistent organizational approach throughout the manuscript.*

We thank the reviewer for the suggestion. We now maintain, in the Decoding part of the Results section, the same organization used for the other parts of this section, without separating the results by anatomical areas.

*- A thorough proofread is recommended to address grammatical errors and instances of unclear or unconventional terminology, which reduce the manuscript's clarity. Some examples include:*

*Abstract: "pragmatic"*

*Introduction: "connectional evidence"*

*Results: "inaction" (easily to be confused with "in action")*

*Caption Figure 2: referring to axes as abscissae and ordinates together with explicit naming of which variables are plotted*

Done.

*- The manuscript inconsistently shifts between present and past tense. The authors should ensure uniformity throughout the text, adhering to the conventional practice of using past tense for describing specific results and present tense for discussing general principles or interpretations*

We corrected this issue, now employing the suggested verbal tense throughout the Result and Discussion section.

*- The figure panels are not described in order in the captions, making it difficult to follow the visual information in a logical sequence. The descriptions should be revised to match the order in which the panels appear*

Done

*- I could not follow the argument in l. 531 ff: it is my understanding that Tanila et al. and the present work both performed a posteriori matching of anatomical and functional data (?)*

This point is now more clearly described in the Introduction and Discussion sections, in which we identify both similarities and differences with the approach used by Tanila and coworkers.
